# Real-Time Noise Removal for Line-Scanning Hyperspectral Devices Using a Minimum Noise Fraction-Based Approach

**DOI:** 10.3390/s150203362

**Published:** 2015-02-03

**Authors:** Asgeir Bjorgan, Lise Lyngsnes Randeberg

**Affiliations:** Department of Electronics and Telecommunications, Norwegian University of Science and Technology, 7491 Trondheim, Norway; E-Mail: lise.randeberg@iet.ntnu.no

**Keywords:** MNF, hyperspectral imaging, real-time, denoising

## Abstract

Processing line-by-line and in real-time can be convenient for some applications of line-scanning hyperspectral imaging technology. Some types of processing, like inverse modeling and spectral analysis, can be sensitive to noise. The MNF (minimum noise fraction) transform provides suitable denoising performance, but requires full image availability for the estimation of image and noise statistics. In this work, a modified algorithm is proposed. Incrementally-updated statistics enables the algorithm to denoise the image line-by-line. The denoising performance has been compared to conventional MNF and found to be equal. With a satisfying denoising performance and real-time implementation, the developed algorithm can denoise line-scanned hyperspectral images in real-time. The elimination of waiting time before denoised data are available is an important step towards real-time visualization of processed hyperspectral data. The source code can be found at http://www.github.com/ntnu-bioopt/mnf. This includes an implementation of conventional MNF denoising.

## 1. Introduction

Push-broom line-scanning hyperspectral cameras can be used for a wide range of applications, e.g., remote sensing, food quality control and waste sorting [1–4]. They have also been used for medical applications, like imaging of human skin, tumor hypoxia and bruises [5–8]. For applications involving rapid scanning, such as objects passing on a conveyor belt or *in vivo* medical diagnostics, it is convenient to do data processing in real-time [9]. A soft real-time requirement is fulfilled when the data processing of a line of data can be constrained to the time window spanned by the arrival of subsequent lines of data. For processing that is sensitive to noise in the measurements, it is imperative to do noise removal as a part of the real-time processing chain.

The maximum noise fraction transform [10] is commonly used for noise removal in hyperspectral imaging. It is a dimensionality reduction technique, where the high amount of data and band-to-band correlations are exploited to extract the pure signal components. The transform is able to efficiently separate signal and noise by reordering the signal space according to increasing SNR (signal-to-noise ratio). This can be used to remove noisy components, while retaining both spectral and spatial resolution. The algorithms presented in our study represent the minimum noise fraction (MNF) transform, which orders the components according to decreasing SNR. There is no difference in denoising performance, only convention.

The image and noise band covariance statistics are used to obtain spectral components with varying levels of noise, which are subsequently used for decomposition. Some cases can make the algorithm less appropriate for denoising: information present only in a few pixels can be dominated by the overall statistics [11]. Substantial changes in contrast can dominate the obtained statistics. Finding the separation between signal and noise can also be a challenging problem to automatize [11–13].

Denoising using the MNF transform is appropriate for hyperspectral imaging in a controlled lab environment. In such settings, the integration time of the hyperspectral camera and the behavior of the light conditions, and thus, the expected levels of noise, are known *a priori*. The boundary between signal and noise can therefore be estimated from prior experience. Close-up reflectance images of human skin are not expected to have large variations in contrast or small regions of spectral anomalies. The images used in this study have a pixel size of approximately 60 × 60 μm and a spatial width of 1600 pixels. Possible deviations are likely to span more than a few pixels (e.g., wounds, arthritic joints).

Real-time processing in a line-scanning setup requires suitable algorithms. The use of MNF for denoising, while appropriate for human tissue imaging, is unsuitable when processing line-by-line. It is assumed that noise and image statistics must be estimated from the image in its entirety, requiring the full image to be available. Other algorithms based on dimensionality reduction schemes [14,15] will typically have the same requirements for image availability. General pixel cleaning techniques, like spatial, or spectral averaging, or binning, will reduce the spectral or spatial resolution.

This paper investigates the use of a modified version of the MNF transform, where the aim is to enable full denoising line-by-line without *a priori* knowledge of image information. It is desired to achieve a similar denoising performance to conventional MNF, due to its advantages and suitability for human tissue imaging. This is challenging, due to the need for proper noise and image covariance matrices in the eigenvalue problem.

The line-by-line MNF (MNF-LBL) was compared against conventional MNF using both simulated and measured hyperspectral data of human skin. The algorithm was implemented in C using matrix operations and library calls to BLAS (Basic Linear Algebra System) [16], ensuring good optimization. The implementation was found to fulfill the real-time requirements of the hyperspectral imaging process, *i.e.*, finish the processing of one line of data within the hyperspectral line acquisition time. This line acquisition time was 30 ms for the experimental setup and camera used in this study (Hyspex VNIR-1600, Norsk Elektro Optikk, Lillestrom, Norway [17]).

A suitably fast line-by-line algorithm for denoising can be useful for scenarios where processing algorithms are to be run line-by-line and the algorithms require a sufficiently noise-free dataset. The application of hyperspectral technology to situations such as these is made possible by the MNF-LBL algorithm. A comparable denoising performance to conventional MNF shows that the method achieves real-time processing feasibility with few tradeoffs.

## 2. Theory

### 2.1. MNF

Assume a hyperspectral image with *P* pixels and *B* bands. The image matrix **Y** is a *B* × *P* matrix defined as

(1) $$Y=\left[{\bar{Y}}_{1},{\bar{Y}}_{2},\ldots ,{\bar{Y}}_{P}\right]$$

The column vector ${\bar{Y}}_{i}^{T}=\left[y_{i,1},y_{i,2},\ldots ,y_{i,B}\right]$ contains the band values associated with the pixel *i*. The noise in the image is assumed to be additive:

(2) Y=S+N

with **S** being the original signal and 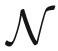 the added noise.

The MNF transformed dataset **T** can be expressed as a linear transformation matrix A operating on the zero-meaned dataset **Z**,

(3) $$T=A^{T}Z$$

The row vectors in the zero-meaned dataset **Z** are found by subtracting the row vectors in **Y** by their means [18]. The first row vectors in **T** contain clean signal components. The rows degrade into pure noise with increasing row number.

It can be shown [10,18] that the transformation matrix **A** = [*ā*_1_,*…*, *ā_b_*] is found by solving the eigenvalue problem

(4) $$\sum_{N}\bar{a}=\lambda \sum \bar{a}$$

The eigenvectors are sorted according to increasing eigenvalues. The matrices Σ_
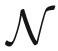
_ and Σ are the covariance matrices of the noise and the image, respectively. The SNR of a particular band *j* in the MNF transformed data can be estimated using [19]

(5) $${\text{SNR}}_{j}=\frac{{\bar{a}}_{j}^{T}\sum {\bar{a}}_{j}}{{\bar{a}}_{j}^{T}\sum_{N}{\bar{a}}_{j}}-1$$

The total fraction *f* of signal conserved in the inverse transformation by using *r* bands can be estimated using

(6) $$f=\frac{\sum_{j=0}^{r-1}{\text{SNR}}_{j}}{\sum_{j}{\text{SNR}}_{j}}$$

The noise matrix 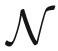 = [*n_b,p_*] is generally not known, but can be estimated using

(7) $$n_{b,p}=\frac{1}{2}\left(y_{b,p}-y_{b,p+1}\right)$$

assuming that neighboring pixels are spatially correlated [18]. Spatial correlation is generally valid for images obtained in remote sensing [10]. It is also valid for high-resolution images of human skin, due to the spectral similarity across the tissue [9].

Keeping only the first *r* high SNR components and skipping the noisy bands in **T** in the inverse transformation results in a denoised image [10,18]. The transformation from the noisy dataset **Z** to a denoised dataset Z* is expressed as [18]:

(8) $$Z^{*}={\left(A^{-1}\right)}^{T}RA^{T}Z$$

(9) =D⋅Z

(10) $$=D\cdot \left[{\bar{Z}}_{1},\ldots ,{\bar{Z}}_{p}\right]$$

where **R** is the identity matrix with the last *B* − *r* diagonal entries set to zero. The number of bands to keep in the inverse transform can be estimated by different methods. The simplest approach is to look at the sorted image bands of the transformed image. The band where no recognizable features can be observed can be chosen as the cut-off band *r* for the inverse transform. It can also be chosen by constraining Equations (5) and (6) to a required SNR and to a required fraction of the preserved signal.

It can be observed that **D** operates separately on each column vector in **Z**. With knowledge of **D**, each pixel can be denoised without any knowledge of the future or previous pixels. Our proposition is to use the current knowledge of the image and noise covariances to estimate the current denoising matrix **D***_i_* and denoise the current set of new pixels **Z***_i_*:

(11) $$Z_{i}^{*}=D_{i}Z_{i}$$

Information is added to the covariances for each new set of pixels. The denoising matrices {**D***_i_*} converge to the denoising matrix **D** as obtained using the whole image at the end of the image. Information necessary for proper denoising is obtained after the few first lines of data.

The above modification to the MNF method can be used to denoise image lines during the image scan without full knowledge of the entire image. This realizes the possibility of performing efficient denoising in real-time during the image scan for line-scanning hyperspectral devices.

### 2.2. Line-by-Line Update of Noise and Image Statistics

The proposed noise removal method requires a new denoising matrix *D_i_* for each new line of data. The statistics required for the derivation of the matrix must therefore be updated line-by-line. The needed statistics is the covariance Σ and the band means *μ̄* = [*μ*_1_,…, *μ_b_*]*^t^*. The latter is needed for the calculation of the former and for estimating the zero-meaned dataset **Z**.

The covariance value at position *i,j* in the covariance matrix is estimated by:

(12) $$\sum_{i,j}=\frac{1}{N}\sum_{p\in \text{P}}\left(y_{i,p}-\mu_{i}\right)\left(y_{j,p}-\mu_{j}\right)$$

where *y_i,p_* is the image value at pixel position *p* and band *i* and *N* is the total number of pixels. This can be expanded to yield:

(13) $$\sum_{i,j}=\frac{1}{N}\sum_{p}y_{i,p}y_{j,p}-\mu_{i}\mu_{j}$$

(14) $$=\frac{1}{N}\sum_{p}y_{i,p}y_{j,p}-\frac{1}{N}\sum_{p}y_{i,p}\frac{1}{N}\sum_{p}y_{j,p}$$

Each of these sums can technically be updated separately line-by-line and combined to yield the current covariance matrix. However, while theoretically correct, this has been found to lead to large numerical errors [20]. Due to the loss of significance in floating point numbers, subtracting one sum from the other may lead to catastrophic cancellation and wrong results [20]. Equation (4) will thus no longer be solvable.

The derivation of numerically stable formulas for combining covariances from different sets can be found in Pebay [21]. These can be exploited for the update of the covariance matrix entries:

(15) $$C_{i,j}^{\text{curr}}=C_{i,j}^{\text{prev}}+C_{i,j}^{\text{line}}+\left(\mu_{i}^{\text{line}}-\mu_{i}^{\text{prev}}\right)\left(\mu_{j}^{\text{line}}-\mu_{j}^{\text{prev}}\right)\frac{N^{\text{line}}\cdot N^{\text{prev}}}{N^{\text{curr}}}$$

(16) $$=C_{i,j}^{*}+\Delta_{i}\Delta_{j}\frac{N^{\text{line}}\cdot N^{\text{prev}}}{N^{\text{curr}}}$$

The line value $C_{i,j}^{\text{line}}$ is found by:

(17) $$C_{i,j}^{\text{line}}=\sum_{p\in \text{L}}\left(y_{i,p}-\mu_{i}^{\text{line}}\right)\left(y_{j,p}-\mu_{j}^{\text{line}}\right)$$

The covariance Σ can then be found using

(18) $$\sum =\frac{1}{N}C$$

The means are updated using

(19) $$\mu_{i}^{\text{line}}=\frac{1}{N^{\text{line}}}\sum_{p\in \text{L}}y_{i,p}$$

and

(20) $$\mu_{i}^{\text{curr}}=\mu_{i}^{\text{prev}}+\frac{N^{\text{line}}}{N^{\text{curr}}}\left(\mu_{i}^{\text{line}}-\mu_{i}^{\text{prev}}\right)$$

Each of Equations (16) and (20) ensure that the updates are done using differences that are not prone to precision issues and catastrophic cancellation [21].

## 3. Experimental Studies

### 3.1. Real-Time Implementation

A line-by-line method for the denoising of hyperspectral images is a prerequisite for real-time denoising of line-scanned hyperspectral images. The method must, however, also be computationally feasible within the real-time deadline limit, in our case of 30 ms, even with other computationally-demanding operations to be scheduled at the same time.

A method for estimating tissue properties in real-time has earlier been developed using GPU (graphics processing unit) programming [9]. This used approximately 3.5 ms of the allotted 30-ms GPU time. For utilization of both GPU and CPU (central processing unit), the denoising method should primarily be implemented using CPU code with the possibility of later extension to the GPU. The update operations have therefore been rewritten as matrix operations and implemented using BLAS [16] and LAPACK (Linear Algebra PACKage) [22], ensuring good memory and cache optimization.

The source code is available for free use under the MIT license at [23]. The code includes both the line-by-line algorithm and a fast implementation of conventional MNF. Details of the implementation can be found in the Appendix.

The algorithms were benchmarked using a computer with an Intel Core i7 CPU (8 cores) and 6 GB of RAM, running Debian GNU/Linux (jessie).

The used BLAS implementations were:

- GSL cblas (version 1.16)
- libatlas (version 3.10.1)
- Intel MKL (version 2013 SP 1.0.080).

Liblapacke (version 3.5.0) was used as the LAPACK implementation. The MNF denoising algorithms were developed using C and linked against the chosen BLAS and LAPACK libraries.

### 3.2. Simulations

**Figure 1.**
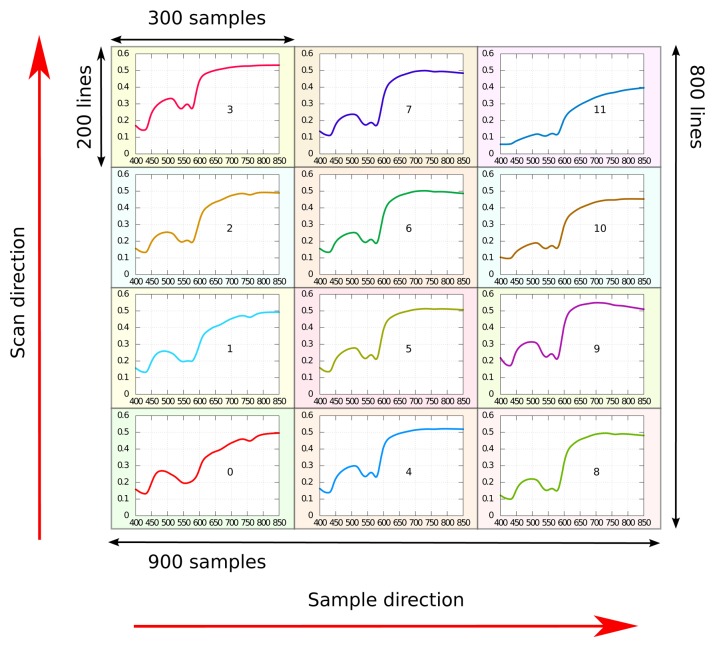
Simulated hyperspectral image. Each region of the image corresponds to a predefined set of optical properties (shown as a plot of the diffuse reflectance within each region).

The new line-by-line MNF was compared against conventional MNF using simulated example data with additive, simulated noise. A hyperspectral image was constructed using known, noise-free reflectance spectra. The image had a dimension of 800 lines × 900 samples × 160 bands. The image was divided into 12 equal-sized blocks, 3 in the sample direction and 4 in the line direction, with all pixels in one block set equal to one sample reflectance spectrum. See Figure 1.

Noise-free example reflectance data were obtained through simulations. A light transport model was used to simulate diffuse reflectance from a model of human skin [24–26]. The skin model was constructed using three homogeneous layers: an epidermis containing melanin and a two-layered dermis containing blood. The skin parameters were varied one at a time in order to generate the necessary 3 × 4 reflectance spectra. An analytic diffusion model [24,27] was used as the light transport model. The simulated spectra are shown in Figure 1.

Noise was added to the image using MATLAB's (version R2013a (8.1.0.604)) imnoise function with zero-meaned, Gaussian noise with constant variance. Three noisy images were generated with different variances: *σ*^2^ = 0.01, *σ*^2^ = 0.001 and *σ*^2^ = 0.0001.

The original, noise-free version of the simulated data was used as a gold standard for benchmarking the output of the noise removal algorithms when applied to the noisy images. SAM (Spectral Angle Mapper) was used to compare the denoised output against the original, noise-free spectra. SAM is defined as the inverse cosine of the Euclidean inner product between two spectra, divided by the Euclidean norm of each spectrum.

### 3.3. Measurements

The methods were compared on measured, noisy hyperspectral data. A hyperspectral image of the dorsal side of the forearm of a 39-year-old female was obtained. The non-skin parts of the image were masked out by using an image subset with a size of 2300 × 1200 pixels.

The image data used were collected using a push-broom HySpex VNIR-1600 camera (Norsk Elektro Optikk, Lillestrom, Norway) [17]. An integration time of 20 μs was used. Two linear light sources were used for illumination (Model 2900 Tungsten Halogen, Illumination Technologies, New York, NY, USA). Polarizers were mounted on the camera lens and the light sources (VersaLight polarizer, VLR-100 NIR, 450–1100 nm, Frederick, CO, USA) in order to avoid specular reflection. A self-developed autofocus system was employed to ensure good focus along the scan axis for the entire scan.

The images were radiometrically calibrated and then converted to reflectance and corrected for uneven illumination across the field of view using a Spectralon reflectance target (SRT-50-050 Reflectance Target, 12.7 x 12.7 cm, ACAL Bfi Nordic AB, Uppsala, Sweden).

An inverse diffusion model [9] was applied to the image in order to estimate blood oxygenation and to compare the results obtained from the noisy and denoised images.

## 4. Results

### 4.1. Noise Removal of Simulated Data

**Figure 2.**
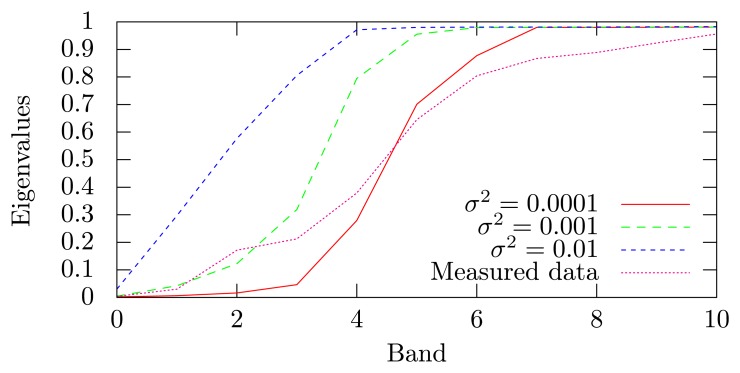
Eigenvalues obtained from the MNF transforms (simulated and experimental data).

The simulated data were denoised using MNF and MNF-LBL by applying the inverse transform on the first *r* bands of the forward transform. The number of bands *r* was chosen differently for each image due to the changing level of noise: *r* = 4 (*σ*^2^ = 0.01, 91.9% of the signal), 5 (*σ*^2^ = 0.001, 98.7% of the signal) and 6 (*σ*^2^ = 0.0001, 99.5% of the signal) of 160 bands, respectively. See the eigenvalue plot in Figure 2 for an estimate of the noise ratio in each band.

**Figure 3.**
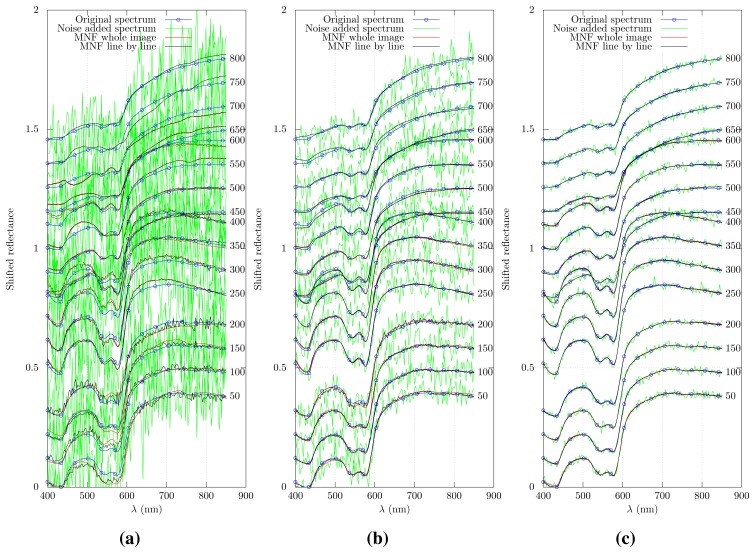
Comparison of simulated and denoised spectra (along *x* = 750). Line numbers are shown to the right. (**a**) *σ*^2^ = 0.01; (**b**) *σ*^2^ = 0.001; (**c**) *σ*^2^ = 0.0001.

Sample sets of the original, simulated spectra, the noise added spectra and the denoised spectra are plotted in Figure 3. Spectra produced using MNF and MNF-LBL overlap after a sufficient number of lines. There is a small difference between the original spectrum and the denoised spectrum for a noise level of *σ*^2^ = 0.0001. This difference becomes more pronounced for increased noise levels. The lines needed for MNF-LBL to accumulate a proper estimate of the noise increase for increasing levels of noise. For *σ*^2^ = 0.0001, *σ*^2^ = 0.001 and *σ*^2^ = 0.01, the required number of lines is approximately 100, 250 and 500, respectively. Before this limit, the noise is reduced instead of removed. The approximate number of lines required for proper denoising was found by visual inspection of the smoothness of the spectra. This could otherwise be calculated by calculating the SNR as a function of line number. However, estimating the SNR as $\frac{\sigma_{\text{Signal}}}{\sigma_{\text{Residual noise}}}$ would likely be sensitive not to the amount of noise, but to the spectral difference between the denoised spectrum and the original spectrum. This would not give the needed information on the effectiveness of the noise-removal, but of how well the denoised spectrum would approximate the original signal.

**Figure 4.**
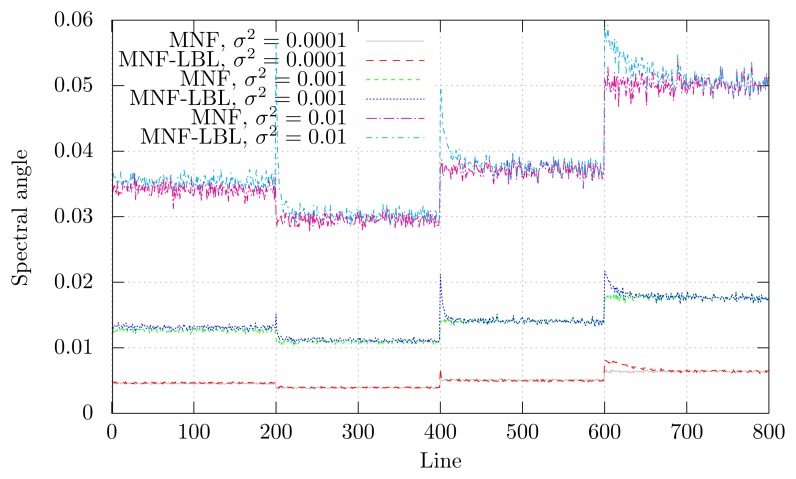
Difference between denoised and simulated spectra.

The difference between the original image and the denoised image is shown as a function of line number in Figure 4 for the column block furthest to the right (*x* > 650). This was calculated using SAM. MNF-LBL produces a higher deviation at a few lines in the beginning of each new region, *i.e.*, every 200 lines. The deviation decreases as the image information is accumulated. MNF and, by extension, MNF-LBL approximate each spectrum using noise-free components. At the beginning of each new region, MNF-LBL approximates these new spectra using the knowledge accumulated from the previous regions. Added information from the new region thus yields a better approximation. The deviations are smaller and have a shorter dampening time with decreasing noise. The differences between MNF-LBL and the original image, and conventional MNF and the original image, are comparable. The other blocks (*x* < 650) did not show any significant differences between MNF and MNF-LBL and did not show significant peaks every 200 lines.

### 4.2. Noise Removal of Measurements

The measured images were denoised using *r* = 8 of 160 bands in the inverse transformation (keeping 99.3% of the signal). This was based on the eigenvalue plot in Figure 2.

**Figure 5.**
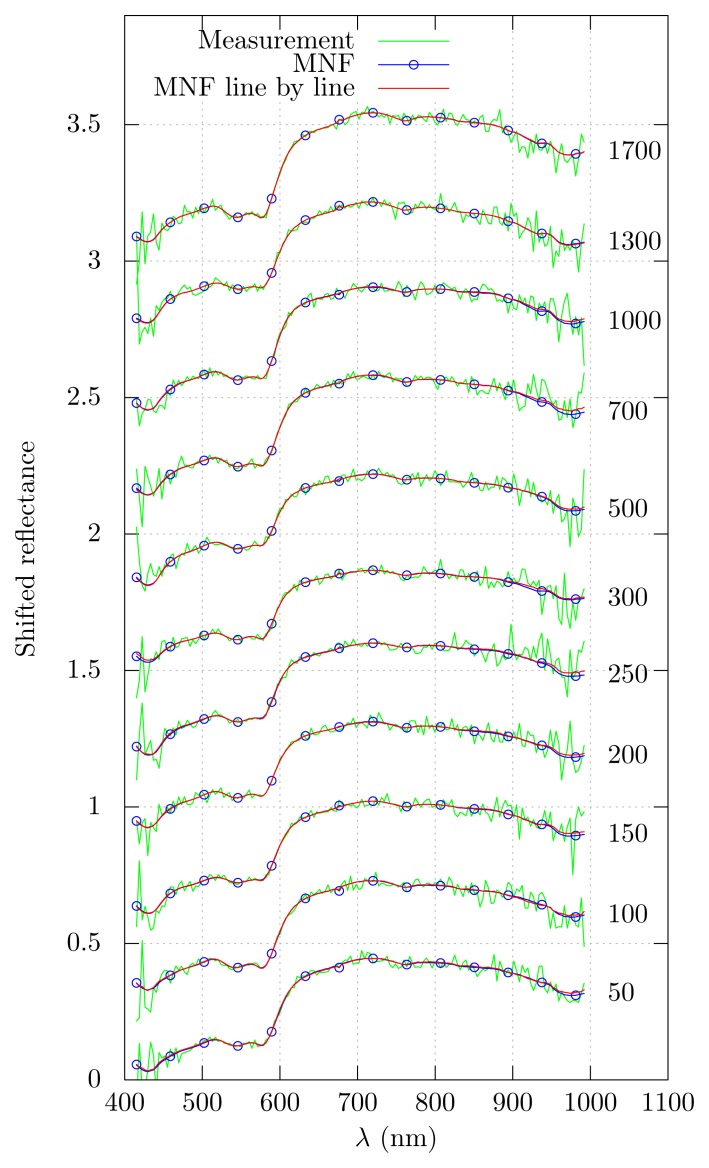
Comparison of denoised spectra along a single image column (experimental data). Corresponding line numbers are shown to the right.

Sample sets of spectra produced using both denoising methods are plotted in Figure 5. MNF-LBL produces a result close to MNF for the low noise parts of the spectra after the first 50 lines of data. The shorter wavelengths are properly denoised after 150 lines. The longer wavelengths need more lines for proper denoising and do not overlap well with the MNF results. This part of the spectrum has a low signal-to-noise ratio, and estimates produced by either method are more prone to error.

**Figure 6.**
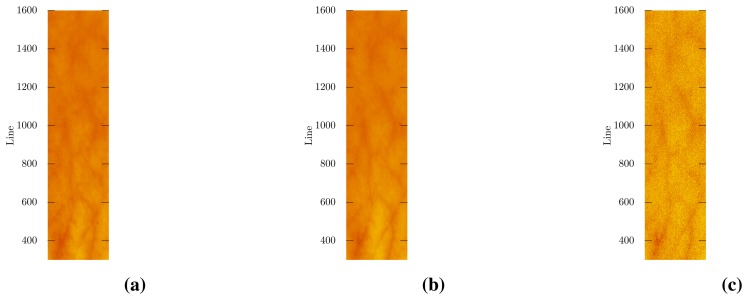
Tissue oxygenation. Inverse diffusion model, wavelength range: 690–820 nm (see [9] for details). (**a**) MNF-LBL; (**b**) MNF; (**c**) Noisy image.

As an example of data processing after noise removal, an inverse diffusion model [9] was applied to the denoised images in order to estimate the blood oxygenation. The results are shown in Figure 6. Both methods produce a noise-free oxygenation map. There are non-substantial differences in the extent of the veins and the exact oxygenation level, which might be due to minute differences in diffuse reflectance spectra and a slight instability in the fitting methods. Inspection of the denoised spectra showed no significant differences in the fitted wavelength ranges (see Figure 5).

### 4.3. Timing

The processing time of the algorithm for a line of hyperspectral data has been measured using different BLAS libraries in order to evaluate the real-time feasibility of the calculations for both open source and commercial libraries, using commodity hardware.

The timing results as run on a line of data with 1600 pixels × 160 wavelengths are shown in Table 1. It is seen that both the ATLAS calls (running on one of eight CPU cores) and Intel MKL calls (running on four of eight CPU cores) finish processing within the real-time deadline limit set by the hyperspectral camera setup (30 ms). Using Intel MKL results in a processing time of 8.5 ms. The remaining processing can be allotted to the remaining four CPU cores, the remaining CPU time on the other four cores and the GPU. This can still guarantee a total processing time of less than 30 ms per line of data. The processing operations can be arbitrarily scheduled, and sequential processing can be run in parallel by doing processing on previous lines.

**Figure 7.**
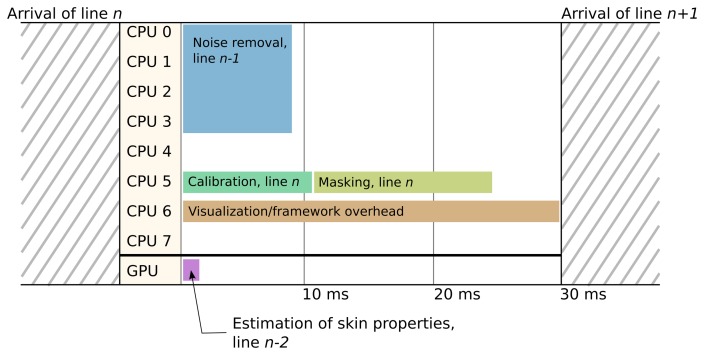
Scheduling of tasks within the real-time framework. The framework overhead time is an example and not an exact measure.

An example of a possible task scheduling is shown in Figure 7. Possible examples of other types of processing not shown here are classification and segmentation.

Calls to BLAS can be exchanged with calls to cuBLAS (CUDA Basic Linear Algebra Subroutines) [28], moving the computations to the GPU. The most time-consuming part, the eigenvalue problem, can be solved every *k*-th line instead of every line.

## 5. Discussion

An algorithm for running noise removal line-by-line on hyperspectral images has been developed using a modification of the minimum noise fraction algorithm. The implementation of this algorithm has shown real-time computational performance. Hyperspectral data have been simulated using known, noise-free reflectance spectra from human skin and by adding Gaussian noise of constant variance. These have been denoised using the conventional MNF transform and the new line-by-line modification and compared to the original, noise-free images. The methods have also been applied to measured human skin data.

MNF-LBL does not provide an advantage over MNF strictly in terms of total computation time. Logically, MNF is faster than MNF-LBL. While conventional MNF only needs to solve the eigenvalue problem once, MNF-LBL must do this once every line. However, the use of MNF requires that all data have been obtained before noise removal can be applied. This limits the applicability of hyperspectral imaging. Some time-critical applications, like medical imaging, would have to wait for the data scan to be finished before noise removal and other processing could be applied. The proposed MNF-LBL algorithm enables noise-free spectra to be obtained during the image scan. Results from subsequent processing can be made available and visualized in real-time. Real-time systems are crucial for this technology to be applicable within diagnostic medicine, where the time limits are strict. For other, more general applications, conventional MNF can still have advantages over MNF-LBL, although the memory requirements of the MNF-LBL are far lower than the requirements of conventional MNF, as only a single pass is made on the data.

For both simulations and measured data, it was shown that MNF-LBL obtains a sufficient noise statistics estimate faster for low noise levels than for high levels of noise. Simulations show that conventional MNF does not reproduce the original spectra accurately for high noise levels. The denoised spectra are smooth, but do not approximate the original spectra. Unphysical artifacts, like minima and peaks, are introduced into the results. Approximately 90% of the signal is kept for the high noise level, as opposed to above 98% for the low noise levels. Any MNF-based method seems therefore to be less suitable for extreme levels of noise. Both methods produce a good estimate of the original spectra for relatively low-noise situations (*σ*^2^ ≈ 0.0001). This is the case for hyperspectral images obtained in a controlled lab setting. The methods appear to have equal performance for the measured data of normal skin. Thus, the trade-off in using MNF-LBL is low for relatively low-noise situations. The trade-off for high-noise situations is evident by the slow convergence of the noise statistics estimate. However, in these cases, the basic MNF method is itself unsuitable.

Hyperspectral images of skin are characterized by small spectral variations across pixels, with few contrast changes. MNF-LBL can be applied to data with a similar behavior. Large and fast changes in contrast might pose challenges. The image covariance will change drastically and can potentially cause sudden changes in the behavior of the denoised image. On the other hand, large contrast variation is an equal burden for conventional MNF and will affect all denoised spectra. The MNF-LBL will only be affected by substantial contrast changes after the change has occurred. One of the strengths of MNF-LBL is that it takes advantage of local image information. This can be exploited by estimating the image covariance in the neighborhood of the current line (or pixel). This will, however, not make it more robust to large contrast changes.

Slow convergence of noise statistics can be alleviated by accumulating lines in the covariance estimate before starting noise removal. Assuming no other processing and a delay of 300 lines, the denoising will catch up with the image scanline after approximately a further 100 lines. This will occur earlier if the eigenvalue problem has to be solved only once. This will still fulfill the real-time needs. No processing time will be needed beyond the time taken to scan the sample. There should be no sharp changes of contrast between the initial 300 lines and the next lines if the denoising is run with incrementally updating covariances after the first 300 lines.

The number of bands *r* in the inverse transform was chosen by visual inspection of the eigenvalue plot and the forward transformed image, as described in Section 2.1. As mentioned in the Introduction, this quantity will generally remain the same for hyperspectral images of skin in the controlled lab setting. For other applications, it may be necessary to automatize the choice by using the alternative method outlined in Section 2.1.

The algorithm has been primarily tested against simulated data with added Gaussian noise of constant variance. The MNF algorithm has earlier been tested for other types of noise [10], and the line-by-line modification will have a similar behavior.

The denoising performance of MNF-LBL is comparable to conventional MNF. With a good, early estimate of the noise statistics, the method gains enough information about the image statistics to reproduce denoised spectra. Conventional MNF produces smooth spectra, while retaining spatial and spectral resolution, but requires the availability of the full image. The presented modification shows that the image availability requirement might not be necessary for obtaining a comparable denoising performance. Thus, the images can be denoised line-by-line. The computational performance of the implementation enables advanced noise removal in real-time during the image scan.

## 6. Conclusions

A real-time line-by-line noise removal algorithm based on the MNF transform has been developed. This algorithm is suitable for line-scanning hyperspectral setups. The algorithm will ensure that noise-free data are available for subsequent processing algorithms in the real-time pipeline. This is an important building block towards real-time medical diagnostic systems based on hyperspectral technology. The algorithm has primarily been tested using biomedical data, but can likely also be used for remote sensing or similar applications. The source code of the method is freely available under an unrestrictive license for testing and further development for other applications.

Minor modifications, like initial line accumulation or neighbor covariance estimation, can be made to improve the method. However, future work will mainly involve using the developed algorithms with other real-time processing routines to create real-time diagnostic systems based on hyperspectral medical imaging.

## Figures and Tables

**Table 1.** Timing results using different BLAS (Basic Linear Algebra System) implementations.

**Version**	**Update Statistics (ms)**	**Solve Eigenvalue Problem (ms)**	**Denoise (ms)**	**Total (ms)**
Unoptimized	116.64	28.43	24.07	169.14
gslcblas	37.56	15.00	28.32	80.88
ATLAS	6.05	11.99	5.11	23.15
MKL	2.38	4.25	1.49	8.12

## Appendix

### A. Real-Time Implementation

The steps of the MNF-LBL algorithm in terms of LAPACK and BLAS operations are outlined here.

#### A.1. Overview

Pixel data are assumed to arrive in separate image line matrices **Y**^line^, defined similarly to Equation (1). The total image matrix is constructed as **Y** = [**Y**^1^,…, **Y***^L^*], where *L* is the total number of lines. The noise ^line^ is estimated using Equation (7).

The vector  = [1,…, 1]*^T^* is an *N*^line^-sized vector consisting only of ones, used for convenience.

The denoising algorithm is implemented using the following steps, line-by-line:

(1) Update statistics (covariances, means)
(2) Solve eigenvalue problem using current statistics
(3) Run denoising using transform matrices obtained from the eigenvalue problem solution

These steps are elaborated below.

#### A.2. Statistics Updates

The mean *μ̄*^line^ is estimated using a matrix-vector multiplication (BLAS: gemv(**Y**, )):

(A1) $${\bar{\mu }}^{\text{line}}=\frac{1}{N^{\text{line}}}Y^{\text{line}}\cdot 1$$

The mean is subtracted from the current line using a rank-one operation (BLAS: ger(-1, *μ̄*, , **Y**)):

(A2) $$Y_{c}^{\text{line}}=-1\cdot {\bar{\mu }}^{\text{line}}\cdot 1^{T}+Y^{\text{line}}$$

The total covariance is updated with the line covariance using a symmetric rank-k operation (BLAS: syrk(**Y**, **C**)):

(A3) $$C^{*}=C^{\text{line}}+C^{\text{prev}}$$

(A4) $$=Y_{c}^{\text{line}}\cdot {\left(Y_{c}^{\text{line}}\right)}^{T}+C^{\text{prev}}$$

The difference Δ̄ and the total mean *μ̄* are found and updated according to Equations (19) and (20). The covariance matrix is updated with the correction term using a symmetric rank-one operation (BLAS: $\text{syr}\left(\frac{N^{\text{line}}\cdot N^{\text{prev}}}{N^{\text{curr}}},\bar{\Delta },C)\right)$:

(A5) $$C^{\text{curr}}=\frac{N^{\text{line}}\cdot N^{\text{prev}}}{N^{\text{curr}}}\cdot \bar{\Delta }\cdot {\bar{\Delta }}^{T}+C^{*}$$

The same updates are run for the noise statistics.

#### A.3. Eigenvalue Problem

The true covariance matrices Σ and Σ__ are found using Equation (18). The LAPACK operation sygv is used to solve the real generalized symmetric-definite eigenproblem in Equation (4). The LAPACK operations getrf and getri are used to find the inverse transformation.

#### A.4. Denoising

The total mean *μ̄*^curr^ is removed from **Y**^line^ using Equation (A2) to yield the zero-meaned dataset **Z**^line^.

The matrix **Z**^line^ is denoised using a series of matrix multiplications (BLAS: gemm) according to Equation (10). The mean is added back to $Z_{*}^{\text{line}}$ to yield the denoised image line using a rank-one operation (BLAS: ger(+1, *μ̄*, , **Z_*_**)):

(A6) $$Y_{*}^{\text{line}}=1\cdot {\bar{\mu }}^{\text{curr}}\cdot 1^{T}+Z_{*}^{\text{line}}$$

The line of image data has now been denoised according to the current knowledge of the image and noise statistics.

## Abbreviations

MNF: minimum noise fraction
MNF-LBL: MNF line-by-line
BLAS: Basic Linear Algebra System
SNR: signal-to-noise ratio
GPU: graphics processing unit
CPU: central processing unit
LAPACK: Linear Algebra PACKage
SAM: Spectral Angle Mapper
cuBLAS: CUDA Basic Linear Algebra Subroutines

## References

[1] Keshava N., Mustard J. (2002). Spectral unmixing. IEEE Signal Process. Mag..

[2] Skauli T., Haavardsholm T.V., KÃěsen I., Arisholm G., Kavara A., Opsahl T.O., Skaugen A. (2010). An airborne real-time hyperspectral target detection system. Proc. SPIE.

[3] Gowen A.A., O'Donnell C.P., Cullen P.J., Downey G., Frias J.M. (2007). Hyperspectral imaging—An emerging process ananalytic tool for food quality and safety control. Trends Food Sci. Technol..

[4] Tatzer P., Wolf M., Panner T. (2005). Industrial application for inline material sorting using hyperspectral imaging in the NIR range. Real Time Imag..

[5] Randeberg L.L., Larsen E.L.P., Svaasand L.O. (2010). Characterization of vascular structures and skin bruises using hyperspectral imaging, image analysis and diffusion theory. J. Biophotonics.

[6] Lu G., Fei B. (2014). Medical hyperspectral imaging: A review. J. Biomed. Opt..

[7] Sorg B.S., Moeller B.J., Donovan O., Cao Y., Dewhirst M.W. (2005). Hyperspectral imaging of hemoglobin saturation in tumor microvasculature and tumor hypoxia development. J. Biomed. Opt..

[8] Randeberg L.L., Hernandez-Palacios J. (2012). Hyperspectral imaging of bruises in the SWIR spectral region. Proc. SPIE.

[9] Bjorgan A., Milanic M., Randeberg L.L. (2014). Estimation of Skin Optical Parameters for Real-Time Hyperspectral Imaging Applications. J. Biomed. Opt..

[10] Green A.A., Berman M., Switzer P., Craig M.D. (1988). A transform for ordering multispectral data in terms of image quality with implications for noise removal. IEEE Trans. Geosci. Remote Sens..

[11] Farzam M., Beheshti S. (2011). Simultaneous denoising and intrinsic order selection in hyperspectral imaging. IEEE Geosci. Remote Sens. Lett..

[12] Cerra D., Muller R., Reinartz P. (2014). Noise Reduction in Hyperspectral Images through Spectral Unmixing. IEEE Geosci. Remote Sens. Lett..

[13] Amato U., Cavalli R.M., Palombo A., Pignatti S., Santini F. (2009). Experimental Approach to the Selection of the Components in the Minimum Noise Fraction. IEEE Geosci. Remote Sens. Lett..

[14] Renard N., Bourennane S., Blanc-Talon J. (2008). Denoising and Dimensionality Reduction Using Multilinear Tools for Hyperspectral Images. IEEE Geosci. Remote Sens. Lett..

[15] Chen G., Qian S.E. (2011). Denoising of Hyperspectral Imagery Using Principal Component Analysis and Wavelet Shrinkage. IEEE Geosci. Remote Sens. Lett..

[16] Dongarra J. (2002). Basic Linear Algebra Subprograms Technical Forum Standard. Int. J. High Perform. Comput. Appl..

[17] Hyspex VNIR-1600. Main Specifications. http://www.hyspex.no/products/hyspex/vnir1600.php.

[18] Gordon C. (2000). A Generalization of the Maximum Noise Fraction Transform. IEEE Trans. Geosci. Remote Sens..

[19] Nielsen A.A., Larsen R. Restoration of GERIS data using the maximum noise fractions transform.

[20] Chan T.F., Golub G.H., LeVeque R.J. (1982). Algorithms for computing the sample variance: Analysis and recommendations. Am. Stat..

[21] Pebay P. (2008). Formulas for Robust, One-Pass Parallel Computation of Covariances and Arbitrary-Order Statistical Moments.

[22] Anderson E., Bai Z., Bischof C., Blackford S., Demmel J., Dongarra J., Du Croz J., Greenbaum A., Hammarling S., McKenney A. (1999). LAPACK Users' Guide.

[23] Bjorgan A., Randeberg L.L. MNF-LBL Source Code. http://github.com/ntnu-bioopt/mnf.

[24] Haskell R.C., Svaasand L.O., Tsay T., Feng T., McAdams M.S., Tromberg B.J. (1994). Boundary conditions for the diffusion equation in radiative transfer. J. Opt. Soc. Am. A..

[25] Spott T., Svaasand L.O. (2000). Collimated Light Sources in the Diffusion Approximation. Appl. Opt..

[26] Randeberg L.L., Winnem A., Haaverstad R., Haugen O.A., Svaasand L.O. (2005). Performance of Diffusion Theory *vs.* Monte Carlo Methods. Proc. SPIE.

[27] Svaasand L., Norvang L., Fiskerstrand E., Stopps E., Berns M., Nelson J. (1995). Tissue parameters determining the visual appearance of normal skin and port-wine stains. Laser Med. Sci..

[28] NVIDIA cuBLAS. https://developer.nvidia.com/cublas.

[29] Iacobus. Project Page. http://www.iacobus-fp7.eu.

